# Is Matrix Gla Protein Associated with Vascular Calcification? A Systematic Review

**DOI:** 10.3390/nu10040415

**Published:** 2018-03-27

**Authors:** Hilary Barrett, Mary O’Keeffe, Eamon Kavanagh, Michael Walsh, Eibhlís M. O’Connor

**Affiliations:** 1 Centre for Applied Biomedical Engineering Research (CABER), School of Engineering, Bernal Institute, University of Limerick, Limerick V94 F858, UK; hilary.barrett@ul.ie (H.B.); michael.walsh@ul.ie (M.W.); 2 School of Natural Sciences and Department of Biological Sciences, University of Limerick, Limerick V94 F858, UK; mary.okeeffe@ul.ie; 3 Department of Vascular Surgery, University Hospital Limerick, Limerick V94 F858, UK; eamon.kavanagh@icloud.com; 4 Health Research Institute (HRI), University of Limerick, Limerick V94 F858, UK; 5 Alimentary Pharmabiotic Centre, Microbiome Institute, University College Cork, Cork T12 YN60, UK

**Keywords:** matrix-Gla-Protein, vascular calcification, vitamin K, cardiovascular disease, atherosclerosis, chronic kidney disease, diabetes, healthy participants

## Abstract

Specific patient cohorts are at increased risk of vascular calcification. Functional matrix-gla protein (MGP), a tissue-derived vitamin K dependent protein, is reported to be an important inhibitor of vascular calcification and may have clinical potential to modify the progression of vascular calcification through regulation of functional MGP fractions. This systematic review examines twenty-eight studies which assess the relationship between circulating protein expressions of MGP species and vascular calcification in different arterial beds. The included studies examined participants with atherosclerosis, chronic kidney disease (CKD), diabetes, healthy participants, vitamin K supplementation, measured plasma vitamin K levels and vitamin K antagonist usage. The current review reports conflicting results regarding MGP fractions with respect to local calcification development indicating that a multifaceted relationship exists between the MGP and calcification. A primary concern regarding the studies in this review is the large degree of variability in the calcification location assessed and the fraction of MGP measured. This review suggests that different underlying molecular mechanisms can accelerate local disease progression within the vasculature, and specific circulating fractions of MGP may be influenced differently depending on the local disease states related to vascular calcification development. Further studies examining the influence of non-functional MGP levels, with respect to specific calcified arterial beds, are warranted.

## 1. Introduction

Abating fatal cardiovascular disease (CVD) events among patients with atherosclerosis, chronic kidney disease, diabetes and the aging population remains an imperative clinical challenge. CVD induces major arterial occlusions and stiffening, which can be largely driven by the development of vascular calcification, and the associated hemodynamic consequences cause high rates of hypertension, myocardial infarction, stroke and lower-limb ischemia [1,2]. The mortality rate of patients with chronic kidney disease (CKD) alone accounts for 50% of premature deaths while additionally patients with peripheral arterial disease are predisposed to lower limb amputations [3,4]. A prognosticator of vascular calcification development is a prerequisite for identifying high risk patients and may have predictive power for either prevention or progression, thereby potentially reducing these catastrophic complications. 

Vitamin K plays an integral role in the regulation of proteins associated with the inhibition of cardiovascular disease related complications [5]. It acts as a cofactor for the enzyme γ-glutamyl carboxylase in the post-translational conversion of glutamic acid (Glu) to γ-carboxyglutamic acid (Gla) residues. This conversion is necessary for the functionality of all vitamin K-dependent proteins (VKDPs) including, matrix-Gla-Protein (MGP), a 14 kDa VKDP, which is secreted primarily by vascular smooth muscle cells (VSMCs) in the arterial medial layer [6] and is considered a potent inhibitor of vascular calcification. Cell culture evidence suggests MGP is also strongly expressed in endothelial cells, from where it is most likely to end up in the circulation since the endothelium has direct contact with the blood. Endothelial cell derived MGP is also thought to play an important role in preventing endothelial mesenchymal transitions that can contribute to the calcification of cells [7], while a lack of MGP also causes arteriovenous malformations (AVMs) [8]. MGP contains five Glu residues and three serine residues, requiring glutamate carboxylation and serine phosphorylation, respectively, to become fully functional [9], and therefore inhibiting arterial calcification development. The identification of the circulating MPG levels may have the clinical potential to attenuate the progression of vascular calcification [10] and provide incremental prognostic information for a cardiovascular related clinical event beyond traditional risk factors [11]. Furthermore, early detection in high risk patients prone to calcification development may act as a useful adjunctive criterion, therefore allowing for early clinical intervention [12]. 

Notwithstanding, there are conflicting reports regarding the exact role of MGP in patients with atherosclerotic disease, CKD and patients taking anticoagulants. It is thought that only functional (γ-carboxylated and serine phosphorylated) MGP can inhibit vascular calcification as low levels of functional MGP have indicated higher levels of vascular calcification in specific patient groups including those suffering from stable ischemic disease [13], diabetes [14,15], long-term oral anticoagulant therapy users [16] and CKD patients [17,18]. Conversely, studies have also demonstrated the potential utility of the non-functional MGP measurements to indicate vascular vitamin K status, cardiovascular disease risk and disease state, including calcification levels, in specific patient cohorts. The measurement of various species of non-functional MGP fractions may act as cardiovascular disease risk and disease markers correlating with future cardiovascular morbidity and mortality [13,19] and also with the extent of prevalent vascular calcification [17,20].

As a consequence of the conflicting evidence, the pathophysiological mechanisms which result in diverse morphological manifestations of disease progression in terms of calcification development in the respective patient cohorts, remain unclear. Therefore, the primary aim of this systematic review was to determine the relationship between MGP and the presence of vascular calcification. 

## 2. Methodology

### 2.1. Search Strategy

**Figure 1:**
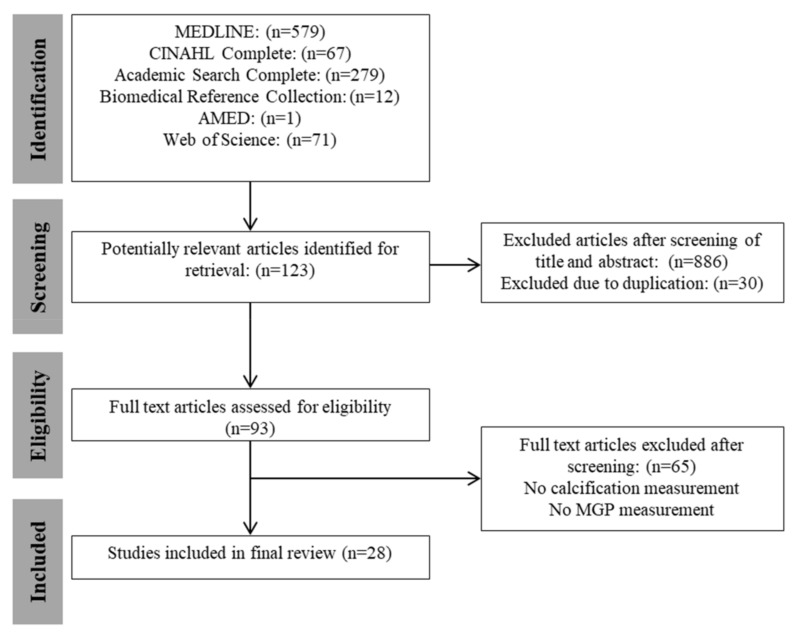
Flow diagram of identification, screening and selection process for included articles in accordance with meta-analysis of observational studies in epidemiology (MOOSE) and preferred reporting items for systematic reviews and meta-analyses (PRISMA). (CINAHL = Cumulative Index to Nursing and Allied Health Literature; AMED = Allied and Complementary Medicine).

This review was registered on the PROSPERO database (CRD 42017084544) and has been reported in accordance with the MOOSE [21] and PRISMA [22] statements. All relevant studies meeting the inclusion criteria were identified by a computer-aided search of Academic Search Complete, AMED, Biomedical Reference Collection, CINAHL, MEDLINE (via EBSCO), and the Web of Science databases during July 2017 from the period of inception (Figure 1). The reference lists of the included manuscripts were searched for additional papers. The search was restricted to include trials that involved humans and were published in English. Two reviewers (H.B. and M.O.) conducted the electronic searches independently. The strategy had two components which were combined: (1) MGP AND (2) calcification. The exact search strings utilized ‘Matrix Gla Protein’ or ‘Matrix Gla-Protein’ or ‘Matrix γ-Carboxyglutamic Acid’ or ‘Matrix Gamma’ or MGP (Abstract) and calcifi* or calcification or calcium or calcified or calcific or ‘coronary artery calcium’ or CAC or mineralization (abstract).

### 2.2. Selection of Studies

Only reports of completed cross sectional, prospective cohort, randomised controlled trials or case control studies in both ex vivo and in vitro conditions published in peer-reviewed journals were included. Studies examining both healthy human subjects and specific human patient cohorts were both included. No restriction was applied to the participant’s age or sex in this analysis. Studies had to report the measurement of a fraction of MGP. Specifically, studies that reported the functional MGP (carboxylated), non-functional MGP (desphosphorylated and carboxylated) and (or) total non-carboxylated were included for review. No restriction was made to the type of MGP measurements performed including both serum/plasma samples through the use of enzyme linked immune assay tests and in the case of the ex vivo studies, performed in the atherosclerosis cohort, immunohistochemistry methods were used. In terms of clinical outcome, the studies had to report results from an outcome measure in the domain of vascular calcification. Studies were not required to have a certain follow-up period.

### 2.3. Study Selection and Data Extraction

A standard protocol was followed for study selection and data extraction. After the removal of duplicates, two authors (H.B. and M.O.) independently screened the titles and abstracts from among the articles found and excluded articles not meeting the eligibility criteria. If no abstract was available, or when it was not clear if the study should be included, full-text articles were retrieved in order to determine inclusion or exclusion. Both reviewers kept a record of their reasons for the inclusion or the exclusion of articles. The full text version of an article was obtained if the title and abstract seemed to fulfil the inclusion criteria or if the eligibility of the study was unclear. 

### 2.4. Risk of Bias Assessment

The methodological quality of included studies was assessed under five domains recommended by the Cochrane Collaboration for assessing risk of bias. The following five domains were considered: (1) study participation and sample size, (2) measurement of risk factor (MGP), (3) measurement of outcome (vascular calcification), (4) statistical analysis and reporting and (5) measurement of and controlling for confounding variables. These domains were chosen to allow for the heterogeneity in study design in this review and due to the lack of an established specific tool for measuring the risk of bias in studies of different designs. Each domain was assessed as having high, moderate or low risk of bias. The overall risk of bias was also assessed. We considered a study to be of low risk of bias when the risk of bias was rated low on at least three of the five domains and was rated low for study confounding. The methodological quality of the included studies was rated independently by two assessors (H.B. and M.O.). The quality assessment scores for all studies are shown in Table 1. 

### 2.5. Data Extraction and Data Analysis

Data regarding each study were extracted and cross-checked by two authors, H.B. and M.O., respectively. The following data were extracted from each study: (1) study type and time of follow-up where applicable (2) characteristics of the study participants (sample size, sex, age, health condition), (3) characteristics of the exposure factor (measure of MGP) (4) characteristics of the outcome (calcification outcome measure) and (5) results summary. Due to substantial heterogeneity across studies, in terms of exposure factors examined, outcome measures used and the length of follow-up, the pooling of data in a meta-analysis was not possible. The findings of each study have been synthesized narratively.

## 3. Results

### 3.1. Literature Search

Study identification is summarised in Figure 1. The literature search of databases yielded 1009 potentially relevant articles of which titles and abstracts were screened. Of this, 123 articles were selected based on their relevance, a total of 30 duplicates were removed, and 93 full text articles were reviewed. From this, 28 full-text studies were retrieved, and 65 studies were excluded as they did not meet the eligibility criteria. Searching the reference lists of these articles did not yield any further articles. Nine authors were emailed regarding their study. No author replied; as a consequence, these studies were considered ineligible.

### 3.2. Risk of Bias Assessment

In total, 13 studies were rated as having a high risk of bias [20,23,24,25,26,28,29,32,33,37,39,40,41,43]. Three studies were rated as having a moderate risk [30,31,34] of bias and 12 were rated as having a low risk of bias [14,15,17,18,27,35,36,38,42,44,45]. A number of common methodological limitations were identified across the studies: low sample size, inappropriate statistical analysis and poor measurement and/or controlling for important confounding factors. Common strengths in the studies were descriptions of the study participants, determinant measurement and outcome measurement. 

### 3.3. Study Characteristics

#### 3.3.1. Study Population

The studies reviewed were designed as randomised controlled trials (*n* = 3) [24,41,44], cross sectional trials (*n* = 23) and longitudinal trials (*n* = 2) [42,43]. In 25 of the articles, the measures of calcification were obtained from in vivo imaging techniques, and in the remaining three studies, calcification was analysed in the arterial tissue in vitro [20,28,32]. Studies examined participants with atherosclerosis (*n* = 6), CKD (*n* = 10), diabetes (*n* = 2), healthy participants (*n* = 4), vitamin K supplementation (*n* = 4), measured plasma vitamin K levels (*n* = 1) and vitamin K antagonist usage (*n* = 1). In the CKD subgroup, Shroff et al. included children on dialysis, and the remaining articles reviewed examined adult patient populations [29].

#### 3.3.2. Calcification Measurement

The calcification was measured in a number of different locations within the vasculature, including the aortic valve (*n* = 5), aorta (*n* = 5), abdominal aorta (*n* = 3), coronary (*n* = 18), carotid (*n* = 3), peripheral arteries (*n* = 4), radial and digital arteries (*n* = 1) and non-specified locations (*n* = 1). The majority of studies quantified the vascular calcification from computed tomography (CT) scans (*n* = 24). Aortic Calcification Severity (AC-24) scores (*n* = 1), Agatston score (*n* = 16), Adragao score (*n* = 2), extended composite scores (*n* = 1), Kauppila scores (*n* = 2) and other total calcification scores (*n* = 2) were also used. In the case of two of the in vitro studies, calcification was detected by Alizarin red von Kossa staining [20,28].

#### 3.3.3. MGP Measurement

The MGP in serum or plasma samples of the patients was measured in terms of four different fractions of MGP, according to the phosphorylation and/or carboxylation processes. The MGP fractions that were analysed included desphosphorylated uncarboxylated (dp-uc) MGP (*n* = 10), total uncarboxylated (t-uc) MGP (*n* = 11), desphosphorylated carboxylated dp-(c) MGP (*n* = 2) and MGP (*n* = 12). The specific MGP fraction concentrations were predominantly measured through the use of enzyme linked immune assay tests. In three articles, the MGP was assessed using immuno-histochemical staining [28,32].

### 3.4. Summary of Study Cohorts

#### 3.4.1. Atherosclerosis

Table 2 presents the six studies which evaluated the relationship between MGP levels and calcification development in patients with atherosclerotic cardiovascular disease which were rated as having high (*n* = 4), moderate (*n* = 1) and low (*n* = 1) risks of bias. Four identified a correlation between MGP and calcification development [20,28,32,34]. In three of these studies, scientific research was performed ex vivo and reported co-localization of non-functional MGP fractions and micro-calcification deposition [20,28,32]. Uncarboxylated MGP has been identified at sites of arterial calcification [20] which has prompted the theory regarding a negative relationship.

#### 3.4.2. Chronic Kidney Disease

Table 3 summarises the ten studies which have reported on the relationship between MGP levels and calcification in CKD patients which were rated as having a high risk of bias (*n* = 5) and low risk of bias (*n* = 5), respectively. Six studies [17,26,29,33,35,36] reported significant correlations between MGP and calcification, while four studies [18,29,39,40] reported a non-significant relationship. For example, Cranenburg et al. identified coronary calcification in end stage renal disease (ESRD) patients whereby vasculature prone to calcify displayed a negative relationship with t-ucMGP levels [36]. 

#### 3.4.3. Vitamin K Antagonists

Rennenberg et al. [30] is the only study included in this review reporting the influence of oral anticoagulants on MGP concentration and calcification development which was rated as having a moderate risk of bias (Table 4). In this study, the average dp-ucMGP levels were significantly higher in coumarin users when compared to patient control levels. Multiple regression analysis further revealed that the use of oral anticoagulants and dp-ucMGP levels were independently associated with the presence of peripheral calcification.

#### 3.4.4. Diabetes

Table 5 summarises the two studies [12,13] which reported the relationship between MGP and vascular calcification in diabetic patients which were both rated as having a low risk of bias. In one study, dp-ucMGP was reported as a positive risk factor for elevated peripheral arterial calcification while t-ucMGP fraction was reported as protective [14]. In the second study, there was a higher odds ratio for the presence of calcification in diabetic patients compared to those without diabetes [15]. 

#### 3.4.5. Healthy Participants

Table 6 summarises the four studies which examined the association between MGP and calcification in healthy subjects and found no correlation in any of the cases. The studies were both rated as having a low risk of bias (*n* = 3) and a moderate risk of bias (*n* = 3). Of these studies, one study [38] reported an association between MGP fractions and calcification levels, whereby lower t-ucMGP levels tended to be associated with lower coronary artery calcification (CAC). The remaining three studies reported no associations between MGP and calcification [27,30,42]. 

#### 3.4.6. Vitamin K Supplementation

Table 7 summarises the five studies [23,24,25,41,43]; of these, four reported on the influence of vitamin K supplementation on MGP concentration and the vascular calcification progression. The studies in this sub group were rated both as having a low risk of bias (*n* = 1) and a high risk of bias (*n* = 4). Of these studies, three [24,41,43] examined vitamin K_2_ supplementation and one [44] examined vitamin K_1_. No studies found an influence on the progression of vascular calcification despite the reduced non-functional fraction of circulating MGP with respect to vitamin K_2_ supplementation. Shea et al. reported that vitamin K_1_ supplementation resulted in a reduction in the rate of calcification progression, as determined by 3-year follow-up imaging to be independent of the total MGP concentration [44]. Within this subgroup, one study reported the levels of both vitamin K_1_ and K_2_ with respect to MGP and calcification and found an association between lower t-ucMGP and CAC [23].

## 4. Discussion

This systematic literature review is the first of its kind to investigate the association between MGP concentration and the presence of vascular calcification in a number of specific patient populations. The review underscores the conflicting results regarding non-functional MGP fractions with respect to local calcification development. From reviewing the current literature, which has evaluated a number of MGP assays in different patient groups at high risk of vascular calcification, it highlights the multifaceted relationship that exists between the two factors. It is not yet clear which MGP species is the most suitable and robust predictor of specific vascular calcification subtypes in given locations for a particular disease state. 

### 4.1. MGP Species

Circulating concentrations and isoforms of vitamin K dependent MGP reaching the circulation depend on the rate of local MGP synthesis, MGP activity in tissue and subsequent binding to calcified areas [16]. In total, four fractions were analysed in the articles reviewed; however, no single MGP species demonstrated a stronger association with vascular calcification. Of the eleven studies that measured t-ucMGP fraction, 54% had a significant correlation with the calcification scores used. This fraction represents the phosphorylated ucMGP containing 1, 2, or 3 phosphoserines as well as a range of degradation products, and thus, it is hypothesised that the negative phospherines have an affinity for binding to the present calcium available [46]. An inverse relationship between the t-ucMGP fraction and calcification has been identified in a number of disease subtypes, including hypertensive patients [30], diabetics [15] and CKD patients [29,36]. A possible explanation for the low concentrations of the non-functional t-ucMGP isoform identified with respect to the presence of calcification in a number of the reviewed studies [29,36] is that messenger-RNA expression does not increase in this high risk cardiovascular disease subtype, resulting in a relative deficiency of MGP secretion and subsequently, predisposing arteries to a high rate of calcification development [25,47]. Moreover, conflicting articles, in which low concentrations of t-ucMGP failed to significantly correlate with calcification, for example, in aortic valve disease [25], underscore the importance of vessel specific associations. 

Dp-ucMGP represents the completely non-functional non-phosphorylated and uncarboxylated form of MGP which has a low affinity for calcium and matrix vesicles and is thus set free into the circulation [17]. Additionally, unlike t-ucMGP, which is not influenced by vitamin K supplementation, high circulating dp-ucMGP levels can reflect a patient’s impaired vitamin K status [48]. Of the ten studies which measured this non-functional MGP fraction, 40% identified an association between MGP and calcification. Among these studies, a strong positive relationship between high levels of dp-ucMGP and vascular calcification in CKD patients [26] was reported among coumarins users [31] and individuals with diabetes [14].

The functional carboxylated MGP fraction forms a high molecular mass complex with calcium phosphate and fetuin [49]. Fetuin is a complementary important circulatory protein that inhibits calcium phosphate crystal precipitation through the presence of this complex, and a deficiency in fetuin protein has been linked with the presence of soft tissue mineralization [50]. In addition to the measurement of the non-functional fractions of MGP, which have been primarily focused on the articles include in this review, it would be useful to isolate the fetuin from the serum in a similar manner to previous approaches [51]. This would provide a complementary adjunct to assess the level of calcification inhibitory action present.

### 4.2. Calcification Development

The complexities of the underlying molecular mechanisms that control calcification development further complicate the interpretation of the relationship with MGP. MGP is synthesized in the tunica media and primarily prevents medial calcification that is predominantly associated with CKD and diabetes [52], whereas atherosclerotic plaque formation and calcification in arteries is predominantly formed at the intimal side, thus suggesting that the complexity of MGP functionality may be specific to each arterial bed and also to each disease state. Multiple mechanisms have been hypothesised as to how functional MGP can inhibit vascular calcification [53,54]. In this review, the calcification of coronary arteries was predominantly assessed in all other vasculature. Coronary artery calcification develops early in the pathogenesis of CVD and is a strong and independent predictor of CVD [55]. While the benefits of this are that it provides standardisation across the different measurements incorporated, certain vasculatures are more prone to developing calcification with respect to the underlying disease condition. For example, it has been reported that subclinical atherosclerosis in a healthy, middle-aged male cohort is most likely identifiable in the femoral arteries [56]. The results of this review imply that the role of MGP may differ between local vascular calcification developments with respect to CVD subtypes. The in vivo calcification measurements were predominantly acquired from CT based imaging and scored by the Agatston method. The ubiquitous use of semi-quantitative metrics, including the Agatston scoring method, should be used with caution when interpreting the level of calcification present, as such a scoring approach is very sensitive to overestimating the calcification burden due to a false high score [57]. It has also been previously demonstrated that the clinical CT resolution constraints partially impede accurate measurements of calcification and delineation from surrounding diseased tissue [58]. Additionally, the fundamental relationships associated with calcification and MGP at the molecular level cannot be truly assessed. As a consequence, the pathophysiological mechanisms, which result in diverse morphological manifestations of disease progression in terms of calcification development in the respective arterial beds, remain unknown [59]. The recent application of molecular imaging techniques in the assessment of arterial calcification have shown promising results in detecting molecular-level metabolic processes association with calcification development [60], and such an approach may help to leverage an improved understanding with respect to MGP’s function.

### 4.3. Study Designs

The majority of articles included were designed as cross sectional studies, assessing the frequency and distribution of vascular calcification with respect to the concentration of different MGP fractions in specific patient cohorts. It is therefore not clear whether the calcification development is solely a consequence of the exposure to non-functional fractions of MGP or whether the calcification progression is derived from other factors that are manifesting concurrently with MGP. Notwithstanding, the benefits of this review is that it highlights the lsrger variation in methods of measurement for both the exposure factor (MGP) and the outcome factor (calcificaiton) and also explores the hypothesis regarding the strength of MGP measurements as a biomarker for calcification and its molecular capability to modify the progression of vascular calcification. 

The clinical trials articles included in this review report on vitamin K supplementation with respect to the progression of vascular calcification and changes in MGP levels have reported conflicting outcomes. The levels of dp-ucMGP significantly decreased in response to vitamin K_2_ supplementation [61]; however, the progression of calcification was independent of the levels [61]. Interestingly, Aoun et al. found a positive correlation between dp-ucMGP and CAC scores at baseline, yet no relationship was found between the decreasing dp-ucMGP levels [41]. It is known that vitamin K_2_ supplementation is a critical cofactor for the carboxylase enzyme responsible for converting the inactive ucMGP to its active form. However, whether vitamin K supplementation can influence the progression of vascular calcification remains unknown. In the case of low vitamin K status, carboxylation is prevented, and thus, vitamin K-dependent proteins, such as MGP, fail to form calcium binding Gla residues. This triggers VSMCs to increase the production of MGP, reflected by an increase in MGP messenger RNA [62]. Healthy reference populations indicate that they are sub-clinically vitamin K deficient and are thus predisposed to vascular calcification due to the loss of calcification inhibition [36].

### 4.4. Future Research and Clinical Implications

While calcification is a significant prognosticator of cardiovascular disease risk, the current review suggests that different underlying molecular mechanisms that accelerate local disease progression exist within the vasculature and that specific circulating fractions of MGP may be influenced differently depending on local disease states related to vascular calcification development. It has been shown that the variation in MGP fractions and the measurement techniques employed preclude firm conclusions and direct comparisons based on the reported data. Future studies need to follow a systematic approach incorporating the established measurement protocols in order to interpret the true value in MGP as a predictor of vascular calcification. Robust prospective studies and randomised controlled trials incorporating multi-centres and a wide range of patients would be very useful to confirm if the differences between patient groups can be similarly identified using the biomarkers described in the current review. Future studies wishing to explore the potential of the vitamin K dependent biomarkers described here should also consider the assessment of dietary vitamin K intake which would help to elucidate the functionality of these biomarkers with respect to vitamin K deficiency and subsequent CVD disease state. Furthermore, it is also very important that future studies control for potential confounders, including age, co-morbidities and medications, which can influence the relationship between MGP and calcification considerably.

### 4.5. Limitations

There are a number of inherent limitations that must be mentioned: (1) only studies published in English were included, and thus relevant studies in other languages might have been excluded. (2) A publication bias may have been introduced as a consequence of the search strategy employed and thus may limit the inclusion of all existing relevant studies which, in some cases, did not meet the predefined inclusion criteria. (3) The majority of studies examined were cross-sectional in nature; thus, the cause of calcification development and progression cannot be inferred from the data presented in these studies. (4) We performed a quality assessment of all studies using the Cochrane criteria, instead of a distinct tool. We acknowledge that this modified approach may have limitations, notwithstanding an established specific tool does not exist to incorporate the presence of various study designs. (5) In the case of full text articles that were not available online, authors were contacted by email. However, in the absence of a response to the request for full text, the study was excluded. 

## 5. Conclusions

In summary, the data generated in this review is a fundamental first step for investigating the association between MGP and vascular calcification. The quantification of the non-functional fractions of MGP advocates their potential for identifying territory specific vascular calcification development in a number of patient cohorts. Notwithstanding, a clear depiction of the associations between MGP and calcification status is partially impeded by the lack of distinction between the function of the different fractions of MGP with respect to the vessel specific calcification analysed in the CVD subtypes. More robust studies with large sample sizes, different populations and thorough controlling for possible confounders are needed. This systematic review therefore advocates the necessity for further investigations into the clinical utility of measuring non-functional MGP to facilitate a better understanding in the early detection of patients at high risk and the mechanisms involved in territory specific arterial calcifications which can result in severe cardiovascular complications.

## Figures and Tables

**Table 1:** Risk of bias analysis classified as low, moderate or high grouped according to patient cohort of specific disease state.

	Study	Study Participation and Sample Size	Risk Factor Measure	Outcome Measure	Statistical Analyses and Reporting	Confounding	Overall Risk of Bias
Athero	[21]	High	Low	Low	Moderate	High	High
	[23]	Low	Low	Low	High	High	High
	[24]	Low	Low	Low	low	Low	Low
	[18]	High	Low	Low	High	High	High
	[25]	Low	Low	Low	low	Moderate	Moderate
	[22]	Moderate	Low	Low	High	High	High
CKD	[26]	High	Low	Low	High	High	High
	[27]	Low	Low	Low	Low	Low	Low
	[28]	Low	Low	Low	High	High	High
	[29]	High	Low	Low	High	High	High
	[30]	Low	Low	Low	Low	Low	Low
	[16]	Low	Low	Low	low	Low	Low
	[15]	Low	Low	Low	Low	Low	Low
	[31]	High	Low	Low	Low	Low	Low
	[32]	Low	Low	Low	High	High	High
	[33]	High	Low	Low	High	High	High
VKA	[34]	High	Moderate	Low	Low	Low	Moderate
Diabetes	[12]	Low	Low	Low	Low	Low	Low
	[13]	Low	Low	Low	Low	Low	Low
Healthy	[35]	Low	Low	Low	Low	Low	Low
	[36]	Low	Low	Low	Low	Low	Low
	[37]	High	Moderate	Low	Low	Low	Moderate
	[38]	Low	Low	Low	Low	Low	Low
VK sup	[39]	Low	Low	Low	High	High	High
	[40]	Low	Low	Low	High	High	High
	[41]	Low	Low	Low	High	High	High
	[42]	Low	Low	Low	Low	Low	Low
	[43]	Low	Low	Low	High	High	High

Athero = atherosclerosis; CKD = chronic kidney disease; VKA; vitamin K Antagonist; and VK supp = vitamin K supplementation. A study was considered to be low risk of bias when the risk of bias was rated low on at least three of the five domains and was rated low for study confounding.

**Table 2:** Association between MGP fractions and vascular calcification in atherosclerotic cardiovascular disease patients.

Author	Study	Population			Outcome Measure			
	Study Design	Cohort	Age (Years)	Sex Male %	Calcification Measurement Method	Calcification Location	MGP Fraction	Main Findings
[32]	Cross sectional	Human autopsy patients (*n* = 6) from non-cardiac causes	47–86 years	NR	3-MeV proton micro beam distribution of micro-calcifications	Coronary	t-ucMGP cMGP Immuno-histochemistry	Micro-calcification correlated with accumulation of t-ucMGP, but not cMGP.
[37]	Cross sectional	Control (*n* = 725); CAW (*n* = 585); AW (*n* = 454); CAP (*n* = 675)	Control 54.9 ± 7.6 CAW 56.8 ± 8.4 AW 57 ± 7.3 CAP 54.2 ± 6.7	Control 70.6% CAW 70.4% AW 70.9% CAP 72.7%	MDCTA 64-slice Agatston score	CAW AW CAP	MGP	A lack of correlation between MGP levels and calcification in any location.
[25]	Cross sectional	Calcific aortic valve disease (*n* = 191) Control (*n* = 35)	71 ± 9 (39–89)	71%	Non-enhanced MSCT 16-slice Agatston score	Aortic valve Coronary	t-ucMGP	No correlation was found between serum t-ucMGP levels and Agatston aortic valve calcification scores in the patient group.
[20]	Cross sectional	Atherosclerotic carotid arteries (*n* = 10), non-diseased carotid artery (*n* = 5) and lower limb arteries (*n* = 6)	73.2 ± 31.47	62%	Alizarin red/Von Kossa staining	Carotid peripheral	MGP t-MGP GluMGP GlaMGP	Advanced carotid plaques (vesicular structures) were present, at the interface of calcium crystal and surrounding tissues mainly co-localizing with GluMGP. In the peripheral arteries, t-MGP was localized in the non-calcified areas and GluMGP was associated with areas of calcification.
[34]	Cross sectional	Patients with stable chest pain/signs of myocardial infarction (*n* = 115)	64 ± 11	60%	EBCT Agatston score	Coronary	MGP	Serum MGP levels were inversely correlated with the severity of coronary CAC scores and found to be independently associated with CAC scores.
[28]	Cross sectional	Autopsy patients (*n* = 10) Atheromatous aortic and coronary artery (*n* = 45) Normal artery (*n* = 4)	44–80	NR	Von Kossa staining	Coronary Aorta	MGP Immunohisto-chemistry	MGP was associated with calcified deposits and sites of early calcification in calciphylaxis and atherosclerosis, and was not detected in normal vessels or in vessels with fibrointimal proliferation.

MGP = matrix Gla protein; t-ucMGP = total uncarboxylated MGP; cMGP = carboxylated MGP; NR = not reported; MDCTA = multidetector computed tomography angiography; EBCT = electron beam computed tomography; MSCT = multislice computed tomography; CAC = cCoronary artery calcification; CAW = coronary artery wall; AW = aortic wall; CAP = coronary atherosclerotic plaque.

**Table 3:** Association between MGP fractions and vascular calcification in chronic kidney disease patients.

Author	Study	Population			Outcome Measure			Main Findings
	Study Design	Cohort	Age (Years)	Sex Male %	Calc Measure Method	Calcification Location	MGP Fraction	
[39]	Cross sectional	Stage V CKD pre dialysis—Balkan endemic nephropathy as primary kidney disease (*n* = 15) and other kidney diseases (*n* = 17)	BEN: 71.7 ± 6.1 Other: 54.7 ± 11.1	BEN: 73% Other: 53%	Radio graphic film Adrago calc score	Iliac Femoral Radial Digital	MGP Immunohistochemistry	No significant difference was found between patients with vascular calcification scores of <4 and ≥4 in the expression of MGP in the wall of the radial artery.
[45]	Cross sectional	ESRD patients (*n* = 97)	45.1 ± 14	64%	MDCT 64-slice scans Agatston score	Coronary	t-ucMGP dp-ucMGP	t-ucMGP and dp-ucMGP levels were not associated with CAC scores.
[26]	Cross sectional	Patient on hemodialysis (*n* = 160) (23 VKA; 137 no VKA)	72 59–81	46%	Lateral X-ray radiography (Kauppila method)	Not specified	dp-ucMGP	dp-ucMGP levels were much higher in patients being treated with VKA, and little overlap was found with those not being treated. dp-ucMGP significantly correlated with calcification score.
[33]	Cross sectional	Patient on hemodialysis (*n* = 64)	60.6 ± 11.3	46.87%	64-slice spiral CT Agatston score	Coronary	MGP	CAC scores were classified into tertiles which revealed a significant positive relationship between increasing CAC and MGP levels.
[35]	Cross sectional	Patient on hemodialysis (*n* = 104); healthy controls (*n* = 14)	Pt. 50.3–56.9 Ctrl. 45.1–64.1	54%	64-row MSCT Agatston score	Coronary Abdominal aorta	MGP	CAC score was significantly associated with MGP but the AAC score was not associated with MGP levels.
[18]	Prospective analysis	Patient on hemodialysis (warfarin excluded) (*n* = 188) Control group (*n* = 98)	Pts. 59 ± 11 Ctrl. 58 ± 15	54%	X-ray/ultrasound Adragao score Extended composite score	Pelvis Hands Av-fistula Carotid mitral aortic valve	dp-ucMGP dp-cMGP	Dp-cMGP levels were not associated with vascular or valvular calcifications at single sites. Using calcification scores, lower dp-cMGP levels correlated with extensive calcification compared with patients with fewer calcifications. Dp-ucMGP levels did not correlate with the extent of vascular calcifications.
[17]	Cross sectional	Caucasian CKD patients (*n* = 107)	67 ± 13	60%	Multi slice spiral CT Kauppila score	Aorta	dp-ucMGP	A positive, statistically significant association was found between the aortic calcium score and plasma dp-ucMGP level.
[36]	Cross sectional	ESRD [CKD stage V] (*n* = 40)	36–87	42.50%	MSCT 16-slice MSCT Agatston score	Coronary aortic valve	t-ucMGP	T-ucMGP levels had a significant association with CAC scores. T-ucMGP levels were significantly lower in patients in the intermediate and high CAC groups in comparison with patients with low CAC scores.
[29]	Cross sectional	Children on dialysis for ≥3 months (*n* = 61)	13.4 ± 4.1	60.60%	16-slice spiral CT Agatston score	Epicardial coronary cardiac valves Aorta	t-ucMGP	T-ucMGP levels in children on dialysis were significantly lower compared to healthy controls but no significant associations were found between t-ucMGP and calcification scores.
[40]	Cross sectional	Patients on hemodialysis (*n* = 30) Renal transplant (*n* = 38)	NR	NR	Quad-slice technique CT total coronary artery calcification score	Coronary aorta	MGP	No correlation was found between MGP levels and calcification of the coronary arteries or aorta. No difference was found in MGP levels between patients with and without calcification.

MGP = matrix Gla protein; t-ucMGP = total uncarboxylated MGP; cMGP = carboxylated MGP; dp-ucMGP = desphosphorylated uncarboxylated; dp-cMGP = desphosphorylated carboxylated; CAC = coronary artery calcification and AAC = abdominal aorta calcification NR = not reported BEN = Balkan endemic nephropathy; ESRD = end stage renal disease; MSCT = multislice computed tomography; VKA = vitamin K antagonist; CKD = chronic kidney disease.

**Table 4:** Association between MGP fractions and vascular calcification in patients taking vitamin K antagonists.

Author	Study	Population			Outcome Measure			Main Findings
	Study Design	Cohort	Age (Years)	Sex Male %	Calc Measure Method	Calc Location	MGP Fraction	
[34]	Cross sectional	Patients on coumarins (*n* = 19) Control (*n* = 18)	Patients 48 (33–56) Control46 (36–53)	64.8%	Soft-tissue 50-kV X-ray based on visibility	Femoral	dp-ucMGP	Coumarin use and dp-ucMGP were associated with femoral artery calcification.

MGP = matrix Gla protein; dp-ucMGP = desphosphorylated uncarboxylated MGP.

**Table 5:** Association between MGP fractions and vascular calcification in diabetic patients.

Author	Study	Population			Outcome Measure			Main Findings
	Study Design	Cohort	Age (Years)	Sex Male %	Calc Measure Method	Calc Location	MGP Fraction	
[14]	Cross sectional	Type 2 diabetics (*n* = 198)	64 ± 8	80%	MSCT 128-slice Agatston score	Popliteal Tibial Peroneal	dp-ucMGP t-ucMGP	dp-ucMGP levels were a positive risk factor for an elevated calcification score and independent predictor of peripheral arterial calcification. t-ucMGP appeared to protect against calcification development.
[15]	Cross sectional	Outpatients with stable CVD (*n* = 839)	68 ± 11	81%	Echocardiography Echo-dense structure	Mitral annular	t-ucMGP	A higher concentration of t-ucMGP was associated with lower odds of MAC in persons without diabetes. A higher concentration of t-ucMGP was associated with higher odds of MAC in persons with diabetes for patients with stable CVD.

MGP = matrix Gla protein; dp-ucMGP = desphosphorylated uncarboxylated MGP; t-ucMGP = total uncarboxylated MGP; MAC = mitral annular calcification; MSCT = multislice computed tomography.

**Table 6:** Association between MGP fractions and vascular calcification in a healthy population cohort.

Author	Study	Population			Outcome Measure			Main Findings
	Study Design	Cohort	Age (Years)	Sex Male %	Calcification Measurement Method	Calc Location	MGP Fraction	
[38]	Cross sectional	Post-menopausal women (*n* = 200)	66.9 ± 5.5	0%	MDCT Agatston score Total volume score	Coronary	t-ucMGP dp-cMGP dp-ucMGP	High t-ucMGP levels were significantly associated with lower CAC. dp-cMGP was not associated with CAC. Low vitamin K-status was associated with high dp-ucMGP concentrations but dp-ucMGP was not associated with CAC in women.
[42]	Cross sectional/Longitudinal	Healthy participants no clinical cardiovascular disease (*n* = 452)	68.25 (66–70)	41%	MSCT 8 slice Agatston score	Coronary	dp-ucMGP	Plasma ucMGP was not associated with CAC in healthy older adults.
[30]	Cross sectional	Hypertensive patients (*n* = 36)	53 ± 10	52.7%	High-resolution CT Agatston score Z-score (total calcium)	Carotid Abdominal aorta Coronary	t-ucMGP	No significant correlation between ucMGP and calc sub scores or total calc z-score. A positive association was identified between total arterial calcium score and lower t-ucMGP.
[27]	Cross sectional	Framingham Offspring Study A: (*n* = 2056) B: (*n* = 452)	A: 57.5 ± 9 B: 68.5 ± 6	A: 51% B: 41%	A: EBCT B: MDCT Agatston score	Coronary	MGP	No association between MGP and CAC after adjustment for CHD risk score in the groups A or B.

MGP = matrix Gla protein; t-ucMGP = total uncarboxylated MGP; cMGP = carboxylated MGP; dp-ucMGP = desphosphorylated uncarboxylated; dp-cMGP = desphosphorylated carboxylated; CAC = coronary artery calcification; EBCT = electron beam computed tomography; MDCT = multidetector computed tomography; MSCT = multislice computed tomography; CHD = coronary heart disease.

**Table 7:** Association between MGP fractions and vascular calcification in patients taking vitamin K supplementation.

Author	Study	Population				Outcome Measure			Main Findings
	Study Design	Cohort	Supplement	Age (Years)	Sex Male %	Calc Measure Method	Calc Location	MGP Fraction	
[41]	Prospective, pre-post intervention clinical trial	Hemodialysis patients (*n* = 50)	360 μg of menaquinone MK-7 once daily	71.50 (med 56.75; IQR 79.25)	30%	X-ray Aortic calcification Severity score (AC-24)	Abdominal aorta	dp-ucMGP	At baseline, dp-ucMGP increased linearly with the increasing calcification score. No correlation between baseline calcification scores and dp-ucMGP drop.
[43]	Longitudinal	Cardiovascular disease (*n* = 26)	15 mg of menaquinone -4 (Vitmain K_2_) 3 times daily	69 ± 8	35%	MSCT 64-slice scanner Agatston score	Coronary	t-ucMGP	CAC significantly increased despite the MK-4 treatment
[24]	Prospective, randomized, and double-blind	Non-dialyzed with CKD stages 3–5 (*n* = 42)	10 μg of cholecalciferol (D *n* = 12)/90 μg (menaquinone, MK-7) with 10 μg of cholecalciferol (K + D *n* = 28)	D: 55.4 ± 15.2 K + D; 59.4 ± 9.6	D; 61.5% K + D; 52%	MSCT Agatston score	Coronary	MGP dp-ucMGP	CAC significantly increased in both groups at the end of treatment period. Vitamin K_2_ does not significantly affect the progression of calc but does significantly change dp-ucMGP levels.
[44]	Double-blind, randomized controlled trial	Healthy men and postmenopausal women subjects (*n* = 388): treatment group (*n* = 200), control group (*n* = 188)	500 µg phylloquinone (Vitamin K_1_)/control group received multivitamin formulation without phylloquinone once daily	68 ± 65	40%	MSCT 8-slice Agatston score	Coronary	MGP	No difference in CAC progression between the phylloquinone group and control group. Neither baseline nor change in MGP concentrations predicted the change in CAC. Phylloquinone supplementation slowed CAC progression older adults with pre-existing CAC, independent of its effect on MGP concentrations.
[23]	Cross sectional	Patients with CVD (*n* = 103)	NR	64 ± 13	57%	MSCT 64-sliceAgatston score	Coronary	t-ucMGP	Coronary CAC score was inversely related to t-ucMGP.

MGP = matrix Gla protein; t-ucMGP = total uncarboxylated MGP; cMGP = carboxylated MGP; dp-ucMGP = desphosphorylated uncarboxylated; CAC = Coronary artery calcification; Calc = calcification; MSCT = multislice computed tomography; CHD = coronary heart disease; CKD = chronic kidney disease; MK = menaquinone NR = not reported.

## References

[1] Wu X.H., Chen X.Y., Fan Y.H., Leung T.W.H., Wong K.S. (2017). High Extent of Intracranial Carotid Artery Calcification Is Associated with Downstream Microemboli in Stroke Patients. J. Stroke Cerebrovasc. Dis..

[2] Zettervall S.L., Marshall A.P., Fleser P., Guzman R.J. (2017). Association of arterial calcification with chronic limb ischemia in patients with peripheral artery disease. J. Vasc. Surg..

[3] London G.M., Guérin A.P., Marchais S.J., Métivier F., Pannier B., Adda H. (2003). Arterial media calcification in end-stage renal disease: Impact on all-cause and cardiovascular mortality. Nephrol. Dial. Transplant..

[4] Guzman R.J., Brinkley D., Schumacher P., Donahue R.M., Beavers H., Qin X. (2008). Tibial artery calcification as a marker of amputation risk in patients with PAD. J. Am. Coll. Cardiol..

[5] Chatrou M.L.L., Reutelingsperger C.P., Schurgers L.J. (2011). Role of vitamin K-dependent proteins in the arterial vessel wall. Hamostaseologie.

[6] Price P.A., Otsuka A.A., Poser J.W., Kristaponis J., Raman N. (1976). Characterization of a gamma-carboxyglutamic acid-containing protein from bone. Proc. Natl. Acad. Sci. USA.

[7] Speer M.Y., Yang H.Y., Brabb T., Leaf E., Look A., Lin W.L., Frutkin A., Dichek D., Giachelli C.M. (2009). Smooth muscle cells give rise to osteochondrogenic precursors and chondrocytes in calcifying arteries. Circ. Res..

[8] Yao Y., Jumabay M., Ly A., Radparvar M., Cubberly M.R., Boström K.I. (2013). A role for the endothelium in vascular calcification. Circ. Res..

[9] Schurgers L.J., Spronk H.M., Skepper J.N., Hackeng T., Shanahan C., Vermeer C., Weissberg P., Proudfoot D. (2007). Post-translational modifications regulate matrix Gla protein function: Importance for inhibition of vascular smooth muscle cell calcification. J. Thromb. Haemost..

[10] Willems B.A.G., Vermeer C., Reutelingsperger C.P.M., Schurgers L.J. (2014). The realm of vitamin K dependent proteins: Shifting from coagulation toward calcification. Mol. Nutr. Food Res..

[11] Dalager S., Falk E., Kristensen I.B., Paaske W.P. (2008). Plaque in superficial femoral arteries indicates generalized atherosclerosis and vulnerability to coronary death: An autopsy study. J. Vasc. Surg..

[12] Ammirati E., Moroni F., Norata G.D., Magnoni M., Camici P.G. (2015). Markers of inflammation associated with plaque progression and instability in patients with carotid atherosclerosis. Mediat. Inflamm..

[13] Mayer O., Seidlerová J., Bruthans J., Filipovský J., Timoracká K., Vaněk J., Černá L., Wohlfahrt P., Cífková R., Theuwissen E. (2014). Desphospho-uncarboxylated matrix Gla-protein is associated with mortality risk in patients with chronic stable vascular disease. Atherosclerosis.

[14] Liabeuf S., Bourron O., Olivier B., Vemeer C., Theuwissen E., Magdeleyns E., Aubert C.E., Brazier M., Mentaverri R., Hartemann A. (2014). Vascular calcification in patients with type 2 diabetes: The involvement of matrix Gla protein. Cardiovasc. Diabetol..

[15] Parker B.D., Schurgers L.J., Vermeer C., Schiller N.B., Whooley M.A., Ix J.H. (2010). The association of uncarboxylated matrix Gla protein with mitral annular calcification differs by diabetes status: The Heart and Soul study. Atherosclerosis.

[16] Schurgers L.J., Joosen I.A., Laufer E.M., Chatrou M.L.L., Herfs M., Winkens M.H.M., Westenfeld R., Veulemans V., Krueger T., Shanahan C.M. (2012). Vitamin K-Antagonists Accelerate Atherosclerotic Calcification and Induce a Vulnerable Plaque Phenotype. PLoS ONE.

[17] Schurgers L.J., Barreto D.V., Barreto F.C., Liabeuf S., Renard C., Magdeleyns E.J., Vermeer C., Choukroun G., Massy Z.A. (2010). The circulating inactive form of matrix Gla protein is a surrogate marker for vascular calcification in chronic kidney disease: A preliminary report. Clin. J. Am. Soc. Nephrol..

[18] Schlieper G., Westenfeld R., Kruger T., Cranenburg E.C., Magdeleyns E.J., Brandenburg V.M., Djuric Z., Damjanovic T., Ketteler M., Vermeer C. (2011). Circulating Nonphosphorylated Carboxylated Matrix Gla Protein Predicts Survival in ESRD. J. Am. Soc. Nephrol..

[19] Ueland T., Dahl C.P., Gullestad L., Aakhus S., Broch K., Skardal R., Vermeer C., Aukrust P., Schurgers L.J. (2011). Circulating levels of non-phosphorylated undercarboxylated matrix Gla protein are associated with disease severity in patients with chronic heart failure. Clin. Sci..

[20] Schurgers L.J., Teunissen K.J.F., Knapen M.H.J., Kwaijtaal M., van Diest R., Appels A., Reutelingsperger C.P., Cleutjens J.P.M., Vermeer C. (2005). Novel conformation-specific antibodies against matrix γ-carboxyglutamic acid (Gla) protein: Undercarboitylated matrix Gla protein as marker for vascular calcification. Arterioscler. Thromb. Vasc. Biol..

[21] Agustina M., Fernando A., Laura G., Luz G., Nancy H.L., Ana M.B., Gustavo H.I., Marta F., Nora V., Silvia I.V. (2016). Women’s preferences and mode of delivery in public and private hospitals: A prospective cohort study. BMC Pregnancy Childb..

[22] Moher D., Liberati A., Tetzlaff J., Altman D.G., Altman D., Antes G., Atkins D., Barbour V., Barrowman N., Berlin J.A. (2009). Preferred reporting items for systematic reviews and meta-analyses: The PRISMA statement. PLoS Med..

[23] Torii S., Ikari Y., Tanabe K., Kakuta T., Hatori M., Shioi A., Okano T. (2016). Plasma phylloquinone, menaquinone-4 and menaquinone-7 levels and coronary artery calcification. J. Nutr. Sci..

[24] Kurnatowska I., Grzelak P., Masajtis-Zagajewska A., Kaczmarska M., Stefańczyk L., Vermeer C., Maresz K., Nowicki M. (2015). Effect of vitamin K2 on progression of atherosclerosis and vascular calcification in non-dialyzed patients with chronic kidney disease stage 3–5. Pol. Arch. Med. Wewn..

[25] Koos R., Krueger T., Westenfeld R., Kühl H.P., Brandenburg V., Mahnken A.H., Stanzel S., Vermeer C., Cranenburg E.C.M., Floege J. (2009). Relation of circulating matrix Gla-protein and anticoagulation status in patients with aortic valve calcification. Thromb. Haemost..

[26] Delanaye P., Krzesinski J.-M., Warling X., Moonen M., Smelten N., Médart L., Pottel H., Cavalier E. (2014). Dephosphorylated-uncarboxylated Matrix Gla protein concentration is predictive of vitamin K status and is correlated with vascular calcification in a cohort of hemodialysis patients. BMC Nephrol..

[27] O’Donnell C.J., Shea M.K., Price P.A., Gagnon D.R., Wilson P.W.F., Larson M.G., Kiel D.P., Hoffmann U., Ferencik M., Clouse M.E. (2006). Matrix Gla protein is associated with risk factors for atherosclerosis but not with coronary artery calcification. Arterioscler. Thromb. Vasc. Biol..

[28] Canfield A.E., Farrington C., Dziobon M.D., Boot-Handford R.P., Heagerty A.M., Kumar S.N., Roberts I.S.D. (2002). The involvement of matrix glycoproteins in vascular calcification and fibrosis: An immunohistochemical study. J. Pathol..

[29] Shroff R.C., Shah V., Hiorns M.P., Schoppet M., Hofbauer L.C., Hawa G., Schurgers L.J., Singhal A., Merryweather I., Brogan P. (2008). The circulating calcification inhibitors, fetuin-A and osteoprotegerin, but not Matrix Gla protein, are associated with vascular stiffness and calcification in children on dialysis. Nephrol. Dial. Transplant..

[30] Rennenberg R.J.M.W., de Leeuw P.W., Kessels A.G.H., Schurgers L.J., Vermeer C., van Engelshoven J.M.A., Kemerink G.J., Kroon A.A. (2010). Calcium scores and matrix Gla protein levels: Association with vitamin K status. Eur. J. Clin. Investig..

[31] Rennenberg R.J.M.W., van Varik B.J., Schurgers L.J., Hamulyak K., Cate H.T., Leiner T., Vermeer C., de Leeuw P.W., Kroon A.A. (2010). Chronic coumarin treatment is associated with increased extracoronary arterial calcification in humans. Blood.

[32] Chatrou M.L.L., Cleutjens J.P., van Vusse G.J.D., Roijers R.B., Mutsaers P.H.A., Schurgers L.J. (2015). Intra-section analysis of human coronary arteries reveals a potential role for micro-calcifications in macrophage recruitment in the early stage of atherosclerosis. PLoS ONE.

[33] Xiao D.M., Wu Q., Fan W.F., Ye X.W., Niu J.Y., Gu Y. (2013). Effect of serum FGF-23, MGP and fetuin-A on calcium-phosphate metabolism in maintenance hemodialysis patients. Hemodial. Int..

[34] Jono S., Ikari Y., Vermeer C., Dissel P., Hasegawa K., Shioi A., Taniwaki H., Kizu A., Nishizawa Y., Saito S. (2004). Matrix Gla protein is associated with coronary artery calcification as assessed by electron-beam computed tomography. Thromb. Haemost..

[35] Pencak P., Czerwieńska B., Ficek R., Wyskida K., Kujawa-Szewieczek A., Olszanecka-Glinianowicz M., Więcek A., Chudek J. (2013). Calcification of coronary arteries and abdominal aorta in relation to traditional and novel risk factors of atherosclerosis in hemodialysis patients. BMC Nephrol..

[36] Cranenburg E.C.M., Brandenburg V.M., Vermeer C., Stenger M., Mühlenbruch G., Mahnken A.H., Gladziwa U., Ketteler M., Schurgers L.J. (2009). Uncarboxylated matrix Gla protein (ucMGP) is associated with coronary artery calcification in haemodialysis patients. Thromb. Haemost..

[37] Wang Y., Chen J., Zhang Y., Yu W., Zhang C., Gong L., Shao L., Lu J., Gao Y., Chen X. (2013). Common genetic variants of MGP are associated with calcification on the arterial wall but not with calcification present in the atherosclerotic plaques. Circ. Cardiovasc. Genet..

[38] Dalmeijer G.W., van der Schouw Y.T., Vermeer C., Magdeleyns E.J., Schurgers L.J., Beulens J.W.J. (2013). Circulating matrix Gla protein is associated with coronary artery calcification and vitamin K status in healthy women. J. Nutr. Biochem..

[39] Petkovic N., Maric R., Gajanin R., Batinic D., Cuk M., Ristic S., Djukanovic L. (2016). Prevalence and risk factors of vascular calcification in pre-dialysis patients with Balkan endemic nephropathy. Srp. Arh. Celok. Lek..

[40] Moe S.M., Reslerova M., Ketteler M., O’Neill K., Duan D., Koczman J., Westenfeld R., Jahnen-Dechent W., Chen N.X. (2005). Role of calcification inhibitors in the pathogenesis of vascular calcification in chronic kidney disease (CKD). Kidney Int..

[41] Aoun M., Makki M., Azar H., Matta H., Chelala D.N. (2017). High Dephosphorylated-Uncarboxylated MGP in Hemodialysis patients: Risk factors and response to vitamin K_2_, A pre-post intervention clinical trial. BMC Nephrol..

[42] Shea M.K., O’Donnell C.J., Vermeer C., Magdeleyns E.J.P., Crosier M.D., Gundberg C.M., Ordovas J.M., Kritchevsky S.B., Booth S.L., Donnell C.J.O. (2011). Circulating Uncarboxylated Matrix Gla Protein Is Associated with Vitamin K Nutritional Status, but Not Coronary Artery Calcium, in Older Adults 1–4. J. Nutr..

[43] Ikari Y., Torii S., Shioi A., Okano T. (2015). Impact of menaquinone-4 supplementation on coronary artery calcification and arterial stiffness: An open label single arm study. Nutr. J..

[44] Shea M.K., Donnell C.J.O., Hoffmann U., Dallal G.E., Dawson-Hughes B., Price P.A., Williamson M.K., Booth S.L., O’Donnell C.J., Ordovas J.M. (2009). Vitamin K supplementation and progression of coronary artery calcium in older men and women. Am. J. Clin. Nutr..

[45] Meuwese C.L., Olauson H., Qureshi A.R., Ripsweden J., Barany P., Vermeer C., Drummen N., Stenvinkel P. (2015). Associations between thyroid hormones, calcification inhibitor levels and vascular calcification in end-stage renal disease. PLoS ONE.

[46] Theuwissen E., Smit E., Vermeer C. (2012). The Role of Vitamin K in Soft-Tissue Calcification. Adv. Nutr..

[47] Lomashvili K.A., Wang X., Wallin R., O’Neill W.C. (2011). Matrix Gla protein metabolism in vascular smooth muscle and role in uremic vascular calcification. J. Biol. Chem..

[48] Herrmann W., Obeid R. (2011). Vitamins in the Prevention of Human Diseases.

[49] Price P.A., Thomas G.R., Pardini A.W., Figueira W.F., Caputo J.M., Williamson M.K. (2002). Discovery of a high molecular weight complex of calcium, phosphate, fetuin, and matrix γ-carboxyglutamic acid protein in the serum of etidronate-treated rats. J. Biol. Chem..

[50] Merx M.W., Schäfer C., Westenfeld R., Brandenburg V., Hidajat S., Weber C., Ketteler M., Jahnen-Dechent W. (2005). Myocardial Stiffness, Cardiac Remodeling, and Diastolic Dysfunction in Calcification-Prone Fetuin-A-Deficient Mice. J. Am. Soc. Nephrol..

[51] Price P.A., Nguyen T.M.T., Williamson M.K. (2003). Biochemical characterization of the serum fetuin-mineral complex. J. Biol. Chem..

[52] Schurgers L.J., Uitto J., Reutelingsperger C.P. (2013). Vitamin K-dependent carboxylation of matrix Gla-protein: A crucial switch to control ectopic mineralization. Trends Mol. Med..

[53] Reynolds J.L., Joannides A.J., Skepper J.N., Mcnair R., Schurgers L.J., Proudfoot D., Jahnen-Dechent W., Weissberg P.L., Shanahan C.M. (2004). Human vascular smooth muscle cells undergo vesicle-mediated calcification in response to changes in extracellular calcium and phosphate concentrations: A potential mechanism for accelerated vascular calcification in ESRD. J. Am. Soc. Nephrol..

[54] Van Gorp R.H., Schurgers L.J. (2015). New insights into the pros and cons of the clinical use of vitamin K antagonists (VKAs) versus direct oral anticoagulants (DOACs). Nutrients.

[55] Criqui M.H., Knox J.B., Denenberg J.O., Forbang N.I., McClelland R.L., Novotny T.E., Sandfort V., Waalen J., Blaha M.J., Allison M.A. (2017). Coronary Artery Calcium Volume and Density: Potential Interactions and Overall Predictive Value: The Multi-Ethnic Study of Atherosclerosis. JACC Cardiovasc. Imaging.

[56] Laclaustra M., Casasnovas J.A., Fernández-Ortiz A., Fuster V., León-Latre M., Jiménez-Borreguero L.J., Pocovi M., Hurtado-Roca Y., Ordovas J.M., Jarauta E. (2016). Femoral and carotid subclinical atherosclerosis association with risk factors and coronary calcium: The AWHS study. J. Am. Coll. Cardiol..

[57] Demer L.L., Tintut Y., Nguyen K.L., Hsiai T., Lee J.T. (2017). Rigor and Reproducibility in Analysis of Vascular Calcification. Circ. Res..

[58] Barrett H.E., Cunnane E.M., O’Brien J.M., Moloney M.A., Kavanagh E.G., Walsh M.T. (2017). On the effect of computed tomography resolution to distinguish between abdominal aortic aneurysm wall tissue and calcification: A proof of concept. Eur. J. Radiol..

[59] Herisson F., Heymann M.-F., Chétiveaux M., Charrier C., Battaglia S., Pilet P., Rouillon T., Krempf M., Lemarchand P., Heymann D. (2011). Carotid and femoral atherosclerotic plaques show different morphology. Atherosclerosis.

[60] Irkle A., Vesey A.T., Lewis D.Y., Skepper J.N., Bird J.L., Dweck M.R., Joshi F.R., Gallagher F.A., Warburton E.A., Bennett M.R. (2015). Identifying active vascular microcalcification by 18F-sodium fluoride positron emission tomography. Nat. Commun..

[61] Kurnatowska I., Grzelak P., Masajtis-Zagajewska A., Kaczmarska M., Stefańczyk L., Vermeer C., Maresz K., Nowicki M. (2016). Plasma Desphospho-Uncarboxylated Matrix Gla Protein as a Marker of Kidney Damage and Cardiovascular Risk in Advanced Stage of Chronic Kidney Disease. Kidney Blood Press. Res..

[62] Shanahan C.M., Proudfoot D., Farzaneh-Far A., Weissberg P.L. (1998). The role of Gla proteins in vascular calcification. Crit. Rev. Eukaryot. Gene Expr..

